# Eucain Lactate

**Published:** 1904-10

**Authors:** 


### Eucain Lactate.

Prof. Katz, of Berlin, (*Therapeutische Monatshefte*, August, 1904,) reports excellent results from the use of eucain lactate in rhinology and otology. This new salt is soluble in water to the extent of 25 per cent., while eucain hydrochlorate is only about 3½ per cent. water solution. A tamponade moistened with 15 per cent. eucain lactate solution and left in the nose for four or five minutes produced a thoroughly satisfactory anesthesia prior to the introduction of the sound for diagnosing the position, size and nature of polypi and nasal hypertrophies. For the removal of hypertrophies by snare or forceps eucain is preferable to cocain, as it is far less toxic and does not cause shrinkage. There is, besides, no secondary hemorrhage, as eucain lactate does not possess the constricting action of cocain. In some cases the new salt was used in 2 per cent. strength for submucosal injection, with prompt anesthetic effect, despite the bleeding produced by the puncture of the needle.

In otology eucain lactate is advantageously used for the removal of polypi and granulations as well as prior to passing the sound. Here also it possesses the merit of not causing shrinkage. When, however, local ischemia of the mucous membrane is desirable, it may be used in combination with suprarenal preparations.

Prof. Katz was highly gratified with the results from a dusting powder of eucain lactate with milk sugar, used after surgical procedures in the nose. Especially useful was it after cauterisation for the prevention of growths on the mucous membrane, the powder being insufflated daily for about six days.

